# The N-Terminally Truncated µ3 and µ3-Like Opioid Receptors Are Transcribed from a Novel Promoter Upstream of Exon 2 in the Human *OPRM1* Gene

**DOI:** 10.1371/journal.pone.0071024

**Published:** 2013-08-12

**Authors:** Sonja Andersen, Cecilie Baar, Torill Fladvad, Eivor Alette Laugsand, Frank Skorpen

**Affiliations:** 1 Department of Cancer Research and Molecular Medicine and European Palliative Care Research Centre, Faculty of Medicine, Norwegian University of Science and Technology (NTNU), Trondheim, Norway; 2 Department of Laboratory Medicine, Children's and Women's Health and European Palliative Care Research Centre, Faculty of Medicine, Norwegian University of Science and Technology (NTNU), Trondheim, Norway; Temple University, United States of America

## Abstract

The human µ opioid receptor gene, *OPRM1*, produces a multitude of alternatively spliced transcripts encoding full-length or truncated receptor variants with distinct pharmacological properties. The majority of these transcripts are transcribed from the main promoter upstream of exon 1, or from alternate promoters associated with exons 11 and 13. Two distinct transcripts encoding six transmembrane domain (6TM) hMOR receptors, µ3 and µ3-like, have been reported, both starting with the first nucleotide in exon 2. However, no mechanism explaining their initiation at exon 2 has been presented. Here we have used RT-PCR with RNA from human brain tissues to demonstrate that the µ3 and µ3-like transcripts contain nucleotide sequences from the intron 1-exon 2 boundary and are transcribed from a novel promoter located upstream of exon 2. Reporter gene assays confirmed the ability of the novel promoter to drive transcription in human cells, albeit at low levels. We also report the identification of a “full-length” seven transmembrane domain (7TM) version of µ3, hMOR-1A2, which also contains exon 1, and a novel transcript, hMOR-1Y2, with the potential to encode the previously reported hMOR-1Y receptor, but with exon Y spliced to exon 4 instead of exon 5 as in hMOR-1Y. Heterologous expression of GFP-tagged hMOR variants in HEK 293 cells showed that both 6TM receptors were retained in the intracellular compartment and were unresponsive to exogenous opioid exposure as assessed by their ability to redistribute or affect cellular cAMP production, or to promote intracellular Ca^2+^ release. Co-staining with an antibody specific for endoplasmic reticulum (ER) indicated that the µ3-like receptor was retained at the ER after synthesis. 7TM receptors hMOR-1A2 and hMOR-1Y2 resided in the plasma membrane, and were responsive to opioids. Notably, hMOR-1A2 exhibits novel functional properties in that it did not internalize in response to the opioid peptide [D-Ala2, N-Me-Phe4, Gly-ol5]enkephalin (DAMGO).

## Introduction

Opioids such as morphine, oxycodone, methadone and fentanyl are widely used in the clinic to treat severe pain. They all exert their analgesic effect through binding to the µ opioid receptor. Unfortunately, their use is also associated with serious side-effects, including sedation, inhibition of gastrointestinal transit, nausea and vomiting, and respiratory depression which occasionally may be life-threatening. Clinicians have long observed that different opioids display different pharmacological properties. This has led to the idea that they may act through distinct opioid receptor mechanisms. Evidence for the existence of subtypes of the µ opioid receptor first came from binding studies in mice and rats [1]–[5], and was later supported by the identification of a series of alternatively spliced mRNAs encoding µ opioid receptor variants with structural differences at both their N- and C-terminal ends [6]–[9]. Different variants may display different expression patterns across brain regions [10], [11] and also differ in efficacy and desensitization properties mediated by different opioids [12]–[14]. However, the function and biological relevance of the splice variants are as yet poorly understood.

The structure of the human *OPRM1* gene is complex. The number of exons identified from the cloning of *OPRM1* transcripts or prediction of putative exons based on comparative genome analysis approaches 20, and a similar number of alternatively spliced hMOR-1 transcripts has been reported. The majority of these variant transcripts are transcribed from the main promoter upstream of exon 1 and contain the same exons 1, 2 and 3 as the classical hMOR-1 [15], followed by alternative exons [16], or an extended exon 3 [17]. Alternative promoters have been identified upstream of exon 11 [18] and exon 13 [19], located upstream of exon 1 and exon 2, respectively. At least three distinct transcripts produced from these alternative promoters, hMOR-1G1, hMOR-1G2 [18] and hMOR-1K [19], are predicted to encode receptor variants with only six transmembrane (TM) domains. Two additional 6TM hMOR variants have been described; the µ3 receptor, which contains exon 2 and parts of exon 3 [20], and hMOR-1W, containing exon 2 and the complete sequence of exon 3. hMOR-1W was originally deposited in GenBank in 2003 by our group (Baar *et al*., GenBank accession no. AY364890), but was later published by Cadet *et al.* [21] and Fricchione *et al.* [22] as “µ3-like receptor”.

How the truncated 5′ end of the µ3 transcript is formed has remained unclear, as no explanation for its initiation at exon 2 has been presented. An important clue came from the characterization of the hMOR-1W transcript, which was shown to contain sequences from the 3′ end of intron 1 joined to exon 2. This observation led us to suggest that the sequences upstream of exon 2 might harbor a previously unrecognized promoter that could be employed in transcription of hMOR-1W. Here, we have extended these studies and show that the transcripts encoding hMOR-1W (µ3-like) and µ3 are both transcribed from a novel promoter upstream of exon 2. We also report the identification of a novel “full length” 7TM version of µ3, which contains exon 1 and which we have termed hMOR-1A2. In addition, a transcript with the potential to encode the previously reported hMOR-1Y receptor [6] was identified. In this transcript, which we have termed hMOR-1Y2, exon Y is joined to downstream exon 4 instead of exon 5 as found in hMOR-1Y.

We have expressed green fluorescent protein (GFP) tagged versions of 7TM and 6TM hMOR variants in HEK 293 human embryonic kidney cells, and studied their subcellular localization and effect on cellular forskolin-induced cAMP levels after opioid exposure. In agreement with previous observations, we find that the 6TM receptors are retained in the intracellular compartment, whereas 7TM receptors are expressed at the plasma membrane. Interestingly, the 7TM version of µ3, hMOR-1A2, exhibited novel functional properties in that it did not internalize in response to [D-Ala2, N-Me-Phe4, Gly-ol5]enkephalin (DAMGO), but still was able to mediate downstream signaling. Our results extend the insight into the complex mechanisms regulating µ opioid receptor diversity.

## Materials and Methods

### RT-PCR

**Figure 1:**
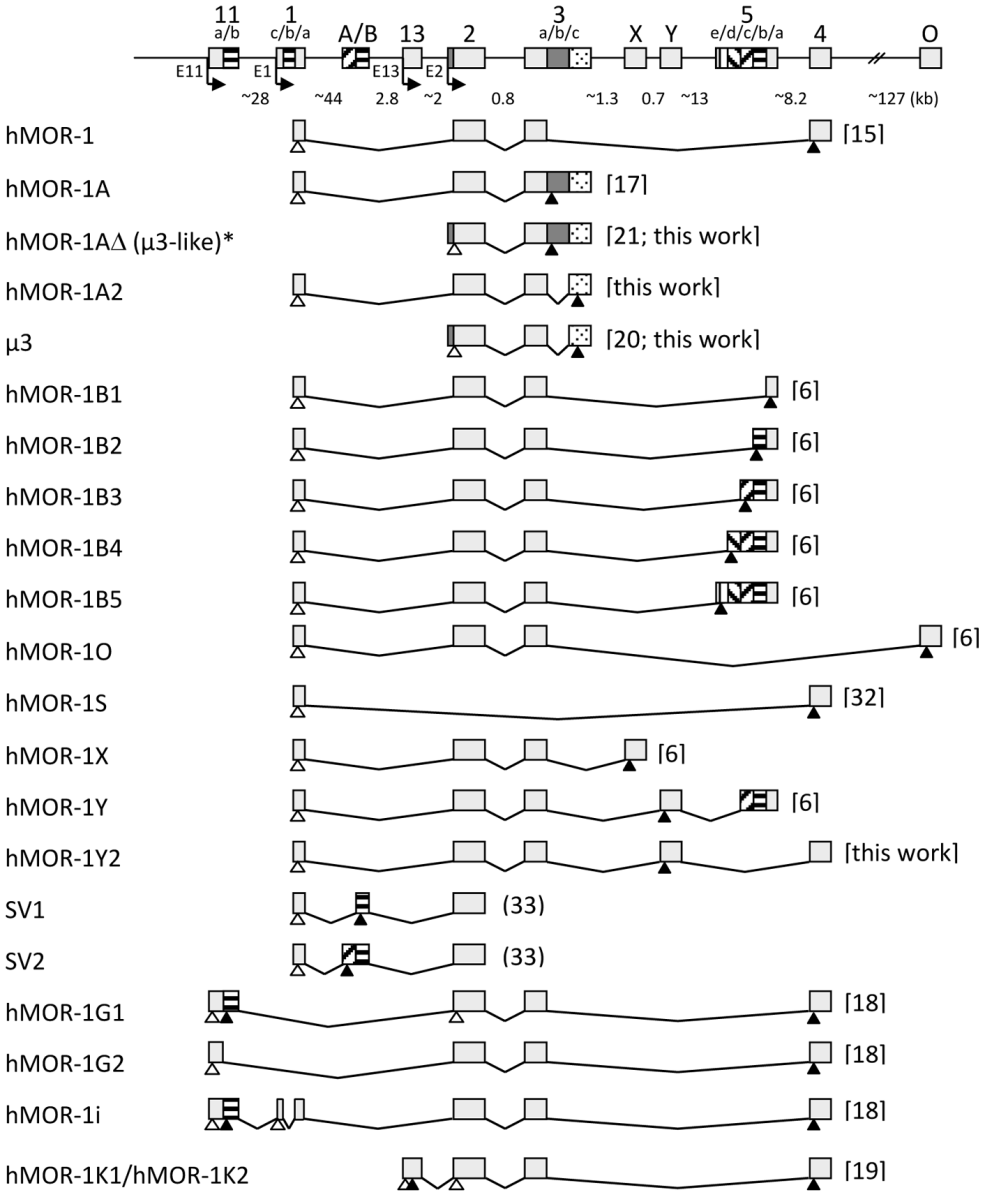
Schematic presentation of *OPRM1* gene structure and alternative splicing. Exons and introns are shown by boxes and horizontal lines, respectively. The approximate sizes of introns (in kb) are indicated. The 5′ extension of exon 2 and the new exon 3B are indicated by grey boxes. The locations of the promoters upstream of exons 11, 1, 13 and 2 are indicated. The transcriptional start sites are indicated by arrows. Putative translation start and stop codons are indicated by open and filled triangles, respectively. For hMOR-1A, the figure includes the full length sequence of hMOR-1A (GenBank accession number NM_001008504.2) which has a longer 3′ UTR than the originally published sequence. This extended sequence was confirmed in the present study (data not shown). All references are commented upon elsewhere in the manuscript, with the exception of the work by Du *et al.* [32] and Choi *et al.* [33]. ^a^ The full length sequence of hMOR-1AΔ (µ3-like) was deposited in GenBank by Baar *et al*. in 2003 as “MOR-1W” (Genbank accession no. AY364890).

**Figure 2:**
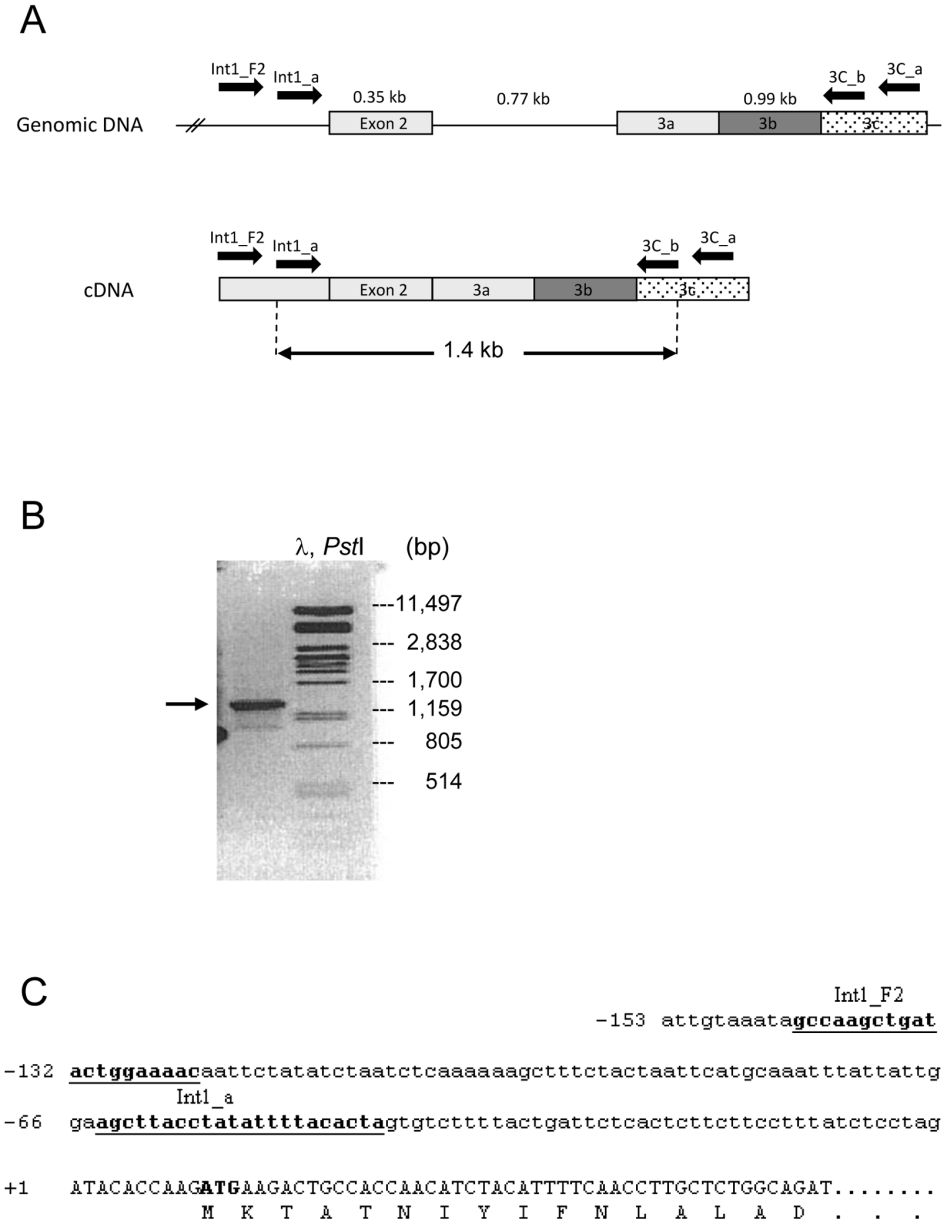
Amplification of the hMOR-1AΔ splice variant originating from the novel E2 promoter. **A.** RNA from human thalamus was used in RT-PCR with forward primers from intron 1 (Int1_F2 and nested Int1_a) and reverse primers from exon 3C (3C_a and nested 3C_b). **B.** The resulting PCR product had a size of about 1400 bp, confirming its origin from cDNA and not genomic DNA (a product originating from genomic DNA would have a predicted size of 2200 bp). **C.** Sequences flanking the intron1-exon2 border. The intron 1 sequence is indicated in lowercase letters and the exon 2 sequence in uppercase letters. The forward primers from intron 1 used in RT-PCR are indicated in bold and are underlined. The putative potential translation start codon (ATG) is indicated in bold.

RT-PCRs were carried out on human brain RNAs (Ambion) isolated from hypothalamus, thalamus, hippocampus, medulla, and amygdala, and from BE(2)-C and SH-SY5Y neuroblastoma cell lines (American Type Culture Collection), using the LightCycler RNA Amplification Kit Master SYBR Green I (Roche). All reactions were performed with 500 ng RNA on a LightCycler System. The oligonucleotide primers (Eurogentec) used in PCR are listed in Table 1. Transcripts originating from the novel E2 promoter were amplified from human thalamus using forward primers from the 3′ end of intron 1 (Int1_F2 and nested Int1_a) along with reverse primers (3C_a and nested 3C_b) from a novel exon in µ3 (here referred to as exon 3C) located 336 bp downstream of exon 3 (here referred to as exon 3A) (Fig. 1 and Fig. 2A). The novel hMOR-1Y2 transcript was amplified by a two-step RT-PCR approach. First, nested RT-PCR was carried out with forward primers from exon Y (Y_c and nested Y_d) and reverse primers from exon 4 (4_a and nested 4_b), which showed that exon Y was directly joined to exon 4. Next, RT-PCR was performed with forward primers from exon 1 (1_a and nested 1_b) in combination with a reverse primer from the exon Y – exon 4 junction (Y/4). The novel hMOR-1A2 transcript was amplified from BE(2)-C cells using forward primers from exon 1 (1_a and nested 1_b) in combination with reverse primers from exon 3C (3C_a and nested 3C_b). PCR products were purified using the QIAquick® PCR Purification Kit, or were recovered from agarose gel using the QIAquick® Gel Extraction Kit (QIAGEN GmbH), and sequenced with the appropriate primers. Complete details on PCR and the cycling conditions used are available on request.

**Table 1:** PCR primers.

Primer	S/A^a^	Exon/position^b^	Sequence
1_a	S	1/33975 – 33994	5′-GCTTGGAACCCGAAAAGTCT-3′
1_b	S	1/34002 – 34020	5′-CCTGGCTACCTCGCACAGC-3′
2_c	A	2/84407 – 84432	5′-CCATTAGGTAATTCACACTCTGGAAG-3′
2_d	A	2/84378 – 84400	5′-GTACTGGTGGCTAAGGCATCTGC-3′
3C_a	A	3C/86417 – 86438	5′-AATATCTTGCATCCATGACCAC-3′
3C_b	A	3C/86370 – 86391	5′-CTTTAATCACAGAACCAGAGCA-3′
4_a	A	4/113315 – 113336	5′-AAAAGCAGGCACTTTCCTAGAG-3′
4_b	A	4/113287 – 113307	5′-GCCTCCTACACATTCTTGAAG-3′
Y_c	S	Y/88735 – 88755	5′-CCAGGGTGTCTGTATTCTGAC-3′
Y_d	S	Y/88758 – 88777	5′-CTGTCCACTGAGGCAATTTC-3′
Y/4	A	Y/4/(88800 – 88818) + (113183 – 113185)	5′-TAGCTTGATAACTGCCAAATCG-3′
Int1_a	S	Int1/84262 – 84283	5′-AGCTTACCTATATTTTACACTA-3′
Int1_F2	S	Int1/84183 – 84203	5′-GCCAAGCTGATACTGGAAAAC-3′
Int1_BglII-4	A	2/TTAGATC + (84338 – 84360)	5′-TTAGATCTGTAGATGTTGGTGGCAGTCTTC-3′
Int1_XmaI-4	S	Int1/CCTCCCGG + (82248 – 82268)	5′-CCTCCCGGGAGAACGGGAAATGAGTGGTT-3′

a : S, sense; A, antisense; ^b^: numbering according to GenBank Accession no. NG_021208.

Restriction sites in Int1_BglII-4 and Int1_XmaI-4 primers are underlined.

### Cloning of the 2 kb region upstream of exon 2

Genomic DNA was used as template in PCR with Int1-BglII-4/Int1-XmaI-4 primers (Table 1), thereby amplifying a 2.1 kb fragment of the sequences upstream of exon 2 (Figure S1). The purified PCR product was given 3′ A-ends using AmpliTaq Gold® polymerase (Applied Biosystems), purified and then inserted into the PCR 2.1-TOPO vector (Invitrogen) containing 3′ T-overhangs. The TOPO-vector with insert was cut with *Kpn*I and *Bgl*II, and the insert was then purified and inserted into the *Kpn*I and *Bgl*II sites of pGL3 Basic (Promega). Several colonies were cultivated, purified, test-cut and one was sequenced and used for further experiments.

### Promoter activity assays

HeLa, SH-SY5Y and BE(2)-C cells were seeded in 48-well plates in 250 µl medium (30–40% confluency), and the following day (at about 70% confluency) the cells were transfected with either 250 ng/well of pGL3 Basic (Promega) or 250 ng/well pGL3 with the putative promoter region using FuGene HD (Promega), 3 µl/µg DNA. pRL-TK (Promega) encoding Renilla luciferase was co-transfected to normalize transfection efficiency (7.5 ng/well). Luciferase activity was measured 24 hours after transfection using the Dual Luciferase Reporter Assay System (Promega). Transfection and luciferase measurements were performed according to the manufacturer's instructions.

### Cloning of hMOR-1 splice variants

Fluorescence-tagged hMOR-1 variants were generated by insertion of PCR amplified cDNAs into the *Hin*dIII/*Xho*I sites of the pcDNA3 vector (Invitrogen) with green fluorescent protein (GFP) already inserted as a C-terminal epitope tag in-frame with an *Xho*I cloning site. The pcDNA3-GFP vector was kindly provided by Dr. Terje Espevik (Institute of Cancer Research and Molecular Medicine, Faculty of Medicine, NTNU). The amplification of hMOR-1 variants for cloning was performed using purified PCR products from the RT-PCRs as template and oligonucleotide primers with integrated *Hin*dIII/*Xho*I restriction sites (Table S1). The antisense primers were designed to either mutate or omit the normal stop-codon in hMOR-1 variants. The PCR reaction was performed with PCR AmpliTaq Gold® polymerase (Applied Biosystems) and all constructs were sequenced to verify the correct sequence.

### Expression of fluorescence-tagged hMOR-1 variants and confocal laser scanning microscopy

Live HEK 293 human embryonic kidney cells (American Type Culture Collection) were seeded on 35 mm glass bottom γ-irradiated tissue culture dishes (MatTek Corp.), and transfected with hMOR/pcDNA3 constructs after 24 h using the FuGENE 6 transfection reagent (Roche). Cells were observed 48 h post-transfection using an Axiovert 100-M confocal microscope (Zeiss), equipped with an LSM 510-META laser-scanning unit and a Zeiss Plan-Apo 63×1.4 numerical aperture oil-immersion objective. Staining (GFP) was visualized using a 488 nm argon laser line and the appropriate filters. For cells exposed to opioids, the culture medium was replaced with phosphate buffered saline (PBS) at 37°C immediately before the addition of either [D-Ala2, N-Me-Phe4, Gly-ol5]enkephalin (DAMGO) (Sigma), morphine (morphine chloride, Lipomed AG), morphine-6-D-glucuronide (M6G) (Lipomed AG), [D-penicillamine2,5]-enkephalin (DPDPE) (Sigma), or sterile water as a negative control.

Stably transfected HEK 293 cells expressing the various hMOR-1 variants were obtained after approximately two weeks of selection with G418 (500 µg/ml). Clones were selected and checked by confocal laser scanning microscopy for expression of GFP-tagged hMOR-1 variants.

### Adenylyl cyclase assay

#### Cell extracts

Adenylyl cyclase activity in living cells was measured as described by Thakker *et al*. [23]. Briefly, two days after seeding, preconfluent stably transfected HEK 293 cells (0.06 – 0.13 mg protein/well) expressing opioid receptors were incubated in Hank's balanced salt solution (HBSS, Sigma, 200 µl/well) with 0.5 mM 3-isobutyl-1-methylxanthine (IBMX) for 5 min at 37°C. After adding HBSS/IBMX with agonist (10 nM – 10 µM) and/or forskolin (10 µM) to a final volume of 500 µl, the plates were incubated at 37°C for 15 min and then placed in boiling water for 3 min before storage at −20°C.

#### cAMP assay

Samples were transferred to Eppendorf tubes, centrifuged (10,000×*g*, 4°C, 5 min) and supernatants placed in new tubes. A competition assay was performed in 24 well plates by mixing 50 µl supernatant, 0.8 pmol [^3^H]-cAMP (diluted in 25 mM Tris-HCl, pH 7 with 0.084% BSA and 0.5 mM IBMX), 75 µl 25 mM Tris-HCl, pH 7 and 15 µl adrenal cortex extract (90 µg). A standard curve was made by using 50 µl of cAMP-solution instead of sample (0.08–50 pmol). After 1 hour incubation on ice, the reaction was terminated by adding 50 µl 50% hydroxyapatite solution and incubating on ice for 6 min. The wells were filled with ice-cold 10 mM Tris-HCl, pH 7 and the samples were immediately harvested on standard glass fiber filter (Packard) on a semi-automatic cell harvester. The filters were washed 6 times, cut, transferred to counting vials and dried. Five ml scintillation fluid (ReadyProtein, Beckman Coulter) was added and the samples were left over night before determining the radioactivity by liquid scintillation counting.

### Immunostaining of transiently transfected cells

Twenty four hours after seeding, HEK 293 cells were transfected using FuGene HD (Roche) and GFP-tagged hMOR-1AΔ (µ3-like) (1 µg plasmid DNA/dish). After 48 hours, cells were washed twice in PBS, fixed in 4% paraformaldehyde for 15 min at RT, washed twice in PBS, permeabilized and blocked by adding PBS with 5% FCS, 2.5% BSA and 0.2% saponin for 45 min at RT. Primary antibody against calnexin (AbCam, ab 13504, an endoplasmic reticulum marker) was diluted 1∶100 in blocking solution (as above) and added to the cells for 45 min at RT. Cells were washed three times in PBS and incubated with secondary antibody (Alexa Fluor 647 goat anti-rabbit, A21244, Invitrogen, 1∶1000 in blocking solution) for 30 min at RT, then washed three times in PBS and stored in PBS at 4°C until examined by confocal laser scanning microscopy (usually the following day).

### Western blots

Total extracts from stably transfected HEK 293 cells expressing four different 7TM-variants of hMOR-1 were made from 50–80% confluent cells being scraped and lysed in a buffer containing 8 M urea, 0.5% Triton-X, 0.1 M DTT, 1x complete protease inhibitor (Roche) and 5x phosphatase inhibitor cocktails 2 and 3 (Sigma). After 2×30 sec of vortexing, samples were centrifuged (15,700×g, 4°C, 15 min) and supernatants snap-frozen in liquid N_2_ and stored at −20°C until gel electrophoresis. Total protein (20–60 µg) was applied to 12% SDS-PAGE gels. After transfer to PDVF membranes, an antibody against GFP (AbCam, Ab290, diluted 1∶2,000) or hMOR-1 (AbCam, Ab137460, 1∶1,000) was added either on membranes from separate gels or on membranes from duplicate wells originating from the same gel (membrane cut in two). Both antibodies were visualized by using a near-infrared (NIR) (800CW) tagged goat anti-rabbit secondary antibody (LiCor; 1∶10,000) and an Odyssey CLx IR scanner. To correct for any variance in loading, an antibody against ß-actin (AbCam, Ab6276, 1∶10,000) was added followed by a NIR (680RD) goat anti-mouse secondary antibody (LiCor, 1∶20,000), and images from the Odyssey Infrared Imaging System were processed using the Li-Cor software. Bands with the expected size (around 72 kDa) being detected by both the GFP antibody and the hMOR-1 antibody, were identified as hMOR-1 splice variants. Results from four western blots were used for determining the relative expression levels of the different receptors.

## Results

### Splice variants hMOR-1AΔ and µ3 are transcribed from a novel promoter upstream of exon 2

In 2003 Cadet *et al.* [20] published the nucleotide sequence of the µ3 opiate receptor (GenBank accession no. AY195733). µ3 is a 6TM receptor which lacks the first of the seven TM domains normally present in G-protein coupled receptors (Figure 1). The study did not, however, provide any information on how the truncated 5′ end of the µ3 transcript, starting with the first nucleotide in exon 2, is formed. Intriguingly, the 5′ end of the µ3 sequence deposited in GenBank (GenBank accession no. AY195733) contains a duplication of the ten first nucleotides of exon 2, which does not conform to the GenBank *OPRM1* gene reference sequence (GenBank accession no. NG021208). To address the possibility that the *OPRM1* gene harbors a previously unrecognized promoter upstream of exon 2, which could be employed in transcription of µ3, a series of RT-PCRs were carried out with RNA derived from human thalamus, utilizing forward primers from the 3′ end of intron 1 along with reverse primers from a novel exon in µ3 (here referred to as exon 3C) located 336 bp downstream of exon 3 (here referred to as exon 3A) (Figure 2, panel A). This resulted in amplification of a 1.4 kb product containing the sequence of the Int1_a forward primer followed by the 42 subsequent 3′-terminal nucleotides of intron 1, and exon 2 directly joined to exon 3A (Figure 2, panels B and C). Moreover, RT-PCRs with primers designed to amplify a region of intron 1 close to the exon 1 border were consistently negative, excluding the possibility that the transcript was derived from incompletely spliced mRNA intermediates. This strongly indicated that the transcript was produced from a novel promoter upstream of exon 2 (from now referred to as the E2 promoter). However, the sequence was distinct from that of µ3, as the 336 bp intervening sequence between exon 3A and exon 3C was retained. The authenticity of the novel transcript was verified in separate RT-PCRs with thalamus RNA from a different donor, as well as with RNA obtained from hypothalamus, hippocampus, medulla, and amygdala (Figure S2, panel A), and from BE(2)-C and SH-SY5Y neuroblastoma cell lines. We denoted this novel variant hMOR-1W and the sequence was deposited in GenBank (Genbank accession no. AY364890). The partial sequence of hMOR-1W was later published by Cadet *et al.* [21] and Fricchione *et al.* [22] and named “µ3-like receptor”, reflecting its similarity to the µ3 receptor published by Cadet *et al.* [20]. The deduced reading frame of hMOR-1W extends 12 nucleotides beyond the normal 3′ end splice site in exon 3A, which makes it similar to hMOR-1A in its C-terminus [17], and we therefore propose that this variant is renamed hMOR-1AΔ instead of “µ3-like” or as originally named hMOR-1W.

Although a PCR product compatible with the expected size of the µ3 transcript (approximately 1.1 kb) was co-amplified with hMOR-1AΔ (µ3-like) from human thalamus using intron 1 primers in combination with exon 3C reverse primers, the band was very weak and we failed to unambiguously demonstrate that the product represented µ3 (Figure 2, panel B). However, a transcript identical to µ3 was amplified using the same primer sets and RNA obtained from BE(2)-C neuroblastoma cells (Figure S2, panel B). This demonstrates that the µ3 variant, like hMOR-1AΔ (µ3-like), can be transcribed from the E2 promoter and is likely to be expressed at very low levels in human brain.

### Identification of a novel 7TM version of µ3, and a novel transcript encoding the previously identified hMOR-1Y

Using forward primers from exon 1 in combination with reverse primers from exon 3C, we amplified a transcript from BE(2)-C cells that was identical to µ3 in its exon combination, but which also contained exon 1 (Figure 1). This transcript may therefore encode a “full length” 7TM version of the µ3 receptor. Except for lacking exon 3B, the structure of this novel transcript is similar to that of hMOR-1A, and we propose that it is named hMOR-1A2. The sequence of hMOR-1A2 has been deposited in GenBank (Genbank accession no. JX914655).

Using RNA from human brain tissues (thalamus, amygdala, medulla) and BE(2)-C and SH-SY5Y neuroblastoma cells, we also amplified a novel transcript with the potential to encode the previously reported hMOR-1Y receptor [6], and which we have termed hMOR-1Y2. Interestingly, in hMOR-1Y2 exon Y is joined to downstream exon 4, instead of exon 5 as in the original hMOR-1Y (Figure 1). This is the only hMOR transcript encoding a full-length receptor in which exon 4 is joined to an upstream exon other than exon 3a. The sequence of hMOR-1Y2 was deposited in GenBank under the accession no. AY364230 (originally deposited as hMOR-1V).

### Analysis of the novel E2 promoter by reporter gene assays

**Figure 3:**
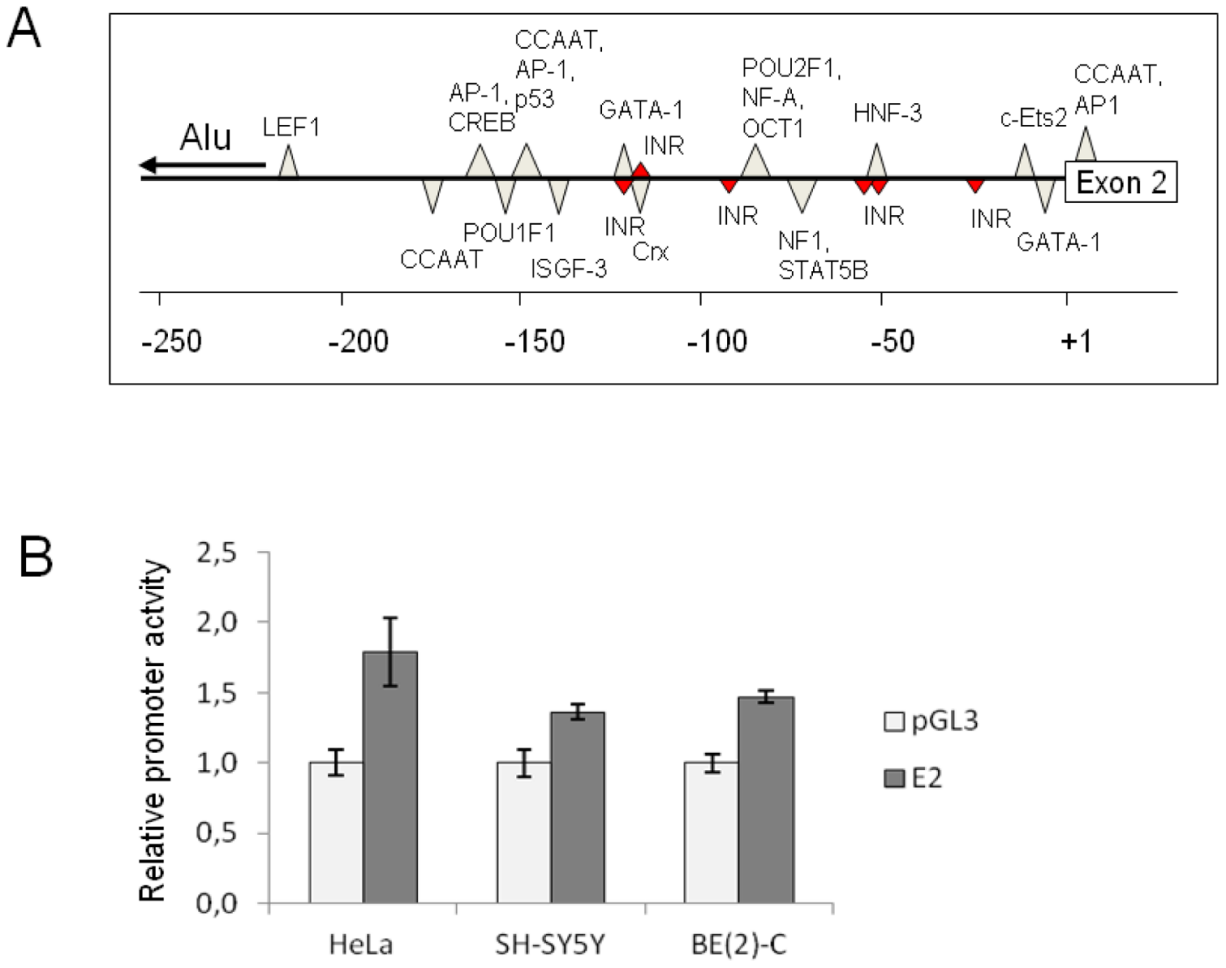
Structure and activity of the E2 promoter. **A.** Schematic presentation of the E2 promoter. Putative transcription factor binding elements are indicated by triangles. The position of an Alu repeat element is indicated by a horizontal arrow. The promoter sequence was examined by the MatInspector and PATCH™ public 1.0 programs for potential binding sites for known transcription factors. **B.** Activity of the E2 promoter assessed by reporter gene assay. HeLa, SH-SY5Y and BE(2)-C cells were transfected with empty pGL3 Basic vector (Promega) or pGL3 containing the putative promoter region, as well as the control vector pRL-TK. Luciferase activity was measured using a dual luciferase kit according to the manufacturer's instructions (Promega). The measurements were performed in two independent experiments with three to six parallels in each. Each experiment comprised three different cell lines and showed comparable results. The results from one representative experiment are shown. The luciferase activity was significantly higher in all cell lines transfected with the novel E2 promoter region as compared to pGL3 Basic vector (p<0.005, student's t-test).

The MatInspector (http://www.genomatix.de/online_help/help_matinspector/matinspector_help.html) and PATCH™ public 1.0, (http://www.gene-regulation.com/cgi-bin/pub/programs/patch/bin/patch.cgi) programs were employed to search for putative binding sites for known transcription factors in a 1 kb region of the 5′ flanking sequence of exon 2. Putative binding sites identified within 250 bp of the exon 2 upstream sequence are shown in Figure 3 (panel A). No element conforming to the consensus TATA box was evident. However, several elements conforming to the initiator element (Inr), which encompasses the transcription start site and has been shown to be sufficient to accurately initiate transcription both *in vitro* and *in vivo* [24], [25] were predicted within 125 bp of the exon 2 immediate 5′ flanking sequence. To test the ability of the 5′ flanking sequence of exon 2 to promote transcription, a chimeric E2-luciferase plasmid containing 2.1 kb of the 5′flanking sequence (Figure S1) was transiently transfected into SH-SY5Y, BE(2)-C, and HeLa cells, and tested for its ability to drive the expression of the luciferase reporter gene. A modest but consistent luciferase production was evident in all three cell lines, suggesting that the cloned sequences do contain the proper combination of activating elements required for transcription (Figure 3, panel B). Luciferase activity was essentially similar between neuronal-like cells and non-neuronal cells, suggesting that the promoter *in vivo* may rely on additional factors or specific cellular conditions for its maximal activity.

### Expression of fluorescence-tagged hMOR-1 variants in HEK 293 cells, and exposure to µ agonists

**Figure 4:**
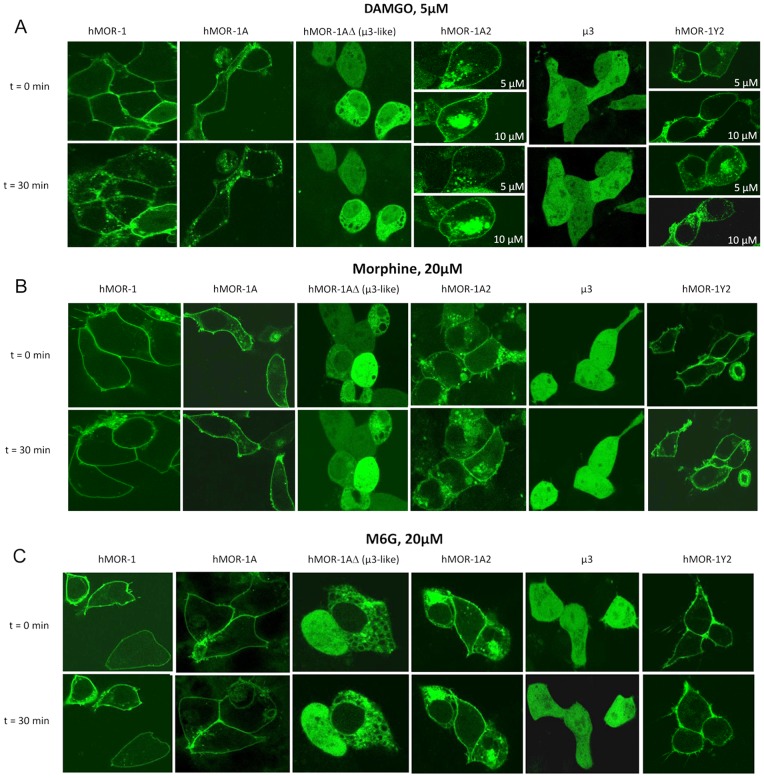
Distribution patterns of C-terminally GFP-tagged hMOR-1 variants transiently expressed in HEK 293 cells. HEK 293 cells were transiently transfected with expression constructs for hMOR-1, hMOR-1A, hMOR-1AΔ (µ3-like), hMOR-1A2, µ3 and hMOR-1Y2 as indicated. In untreated cells (t = 0), hMOR-1, hMOR-1A and hMOR-1Y2 receptors were localized predominantly in the plasma membrane, whereas the novel variant hMOR-1A2 was observed both in the plasma membrane and in intracellular granula. The truncated splice variants hMOR-1AΔ (µ3-like) and µ3 were retained largely intracellularly, either spread throughout the entire cell (like most cells expressing µ3) or associated with intracellular structures and excluded from the nucleus as for many of the cells expressing hMOR-1AΔ (µ3-like). In cells exposed to DAMGO (**A**), hMOR-1, hMOR-1A and to some extent hMOR-1Y2 receptors were observed in numerous intracellular vesicles. No clear effect of DAMGO treatment (5 and 10 µM) was observed with the novel hMOR-1A2. For the truncated receptors (hMOR-1AΔ and µ3), exposure to DAMGO had no effect on receptor localization. Exposure to morphine (**B**) or M6G (**C**) did not affect the intracellular distribution of receptors. Fields of cells were analyzed by confocal microscopy as described in Materials and Methods.

Heterologous expression experiments of fluorescence-tagged hMOR variants were undertaken to analyze their intracellular distribution and their responsiveness to µ-specific agonists. hMOR-1, hMOR-1A, hMOR-1AΔ (µ3-like), hMOR-1A2, µ3 and hMOR-1Y2 were tagged by fusion of GFP to the C-terminal tail. The receptor variants were transiently expressed in HEK 293 cells and analyzed by confocal laser scanning microscopy. Transient expression of GFP-tagged hMOR-1, hMOR-1A, hMOR-1A2 and hMOR-1Y2 revealed that these 7TM receptors resided largely at the plasma membrane (Figure 4), although for hMOR-1A2 significant staining could also be seen as intracellular spots (Figure 4, panels A, B and C, t = 0).

The 6TM hMOR-1AΔ (µ3-like) and µ3 variants were mainly localized intracellularly, either distributed evenly throughout the intracellular compartment including the nucleus (µ3 and the majority of hMOR-1AΔ cells), or receptors were excluded from the nucleus and associated with intracellular structures (some of the hMOR-1AΔ cells) (Figure 4, panels A, B and C).

Exposure to 5 µM of the µ opioid peptide agonist [D-Ala2, N-Me-Phe4, Gly-ol5]enkephalin (DAMGO) for 30 min was accompanied by a clear reduction in membrane labeling and increased intracellular accumulation of hMOR-1 and hMOR-1A, indicating agonist-induced endocytosis (Figure 4, panel A). Internalization was less evident for hMOR-1Y2 at 5 µM DAMGO, but increasing the concentration to 10 µM resulted in pronounced internalization (Figure 4, panel A). In contrast, no detectable internalization was observed for the novel 7TM hMOR-1A2 variant at 5 µM or 10 µM DAMGO (Figure 4, panel A). Exposure to 20 µM morphine or M6G failed to promote detectable internalization of any of the receptors localized at the plasma membrane (Figure 4, panels B and C, respectively). No change in localization was observed with hMOR-1AΔ (µ3-like) and µ3 upon exposure to DAMGO, morphine, or M6G (Figure 4, panels A, B and C). Exposure to the δ agonist [D-Ala2, N-Me-Phe4, Gly-ol5]enkephalin (DPDPE) did not affect receptor localization for any of the splice variants (data not shown).

### hMOR-1AΔ (µ3-like) associates with intracellular structures resembling endoplasmic reticulum

**Figure 5:**
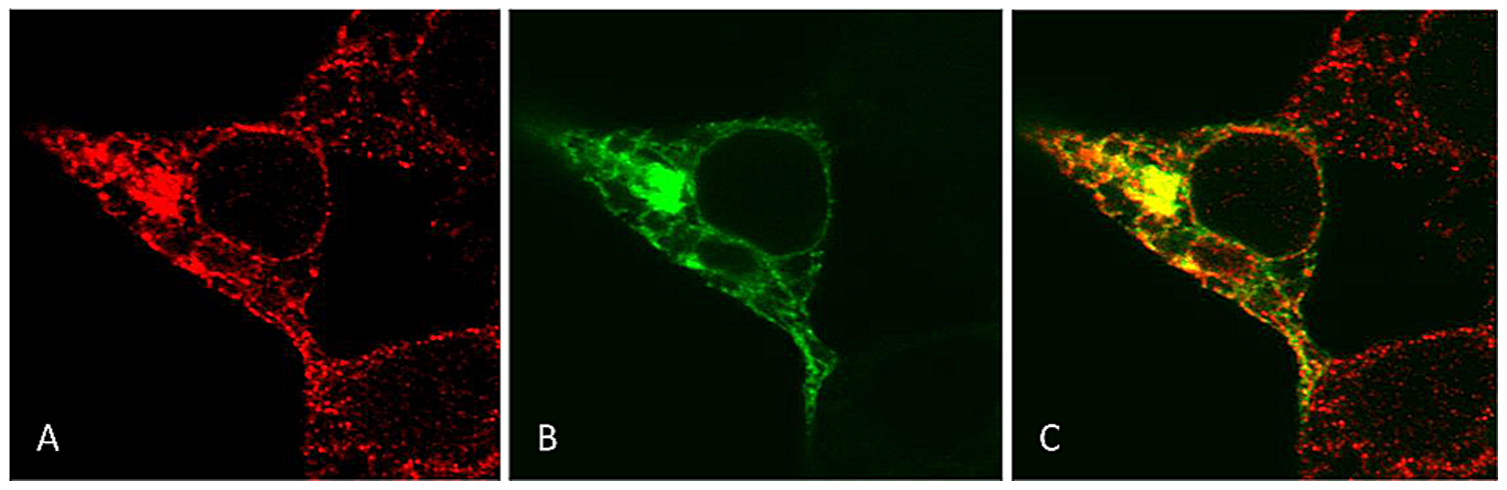
hMOR-1AΔ is associated with endoplasmic reticulum. Transiently transfected HEK 293 cells expressing GFP-tagged hMOR-1AΔ (µ3-like) were fixed in paraformaldehyde and stained with an antibody against endoplasmic reticulum. The staining pattern caused by antibody (**A**) and the receptor itself (**B**) demonstrates a high degree of overlap, shown as yellow color in **C**. This strongly indicates that in cells with this staining pattern, hMOR-1AΔ (µ3-like) is associated with the endoplasmic reticulum.

In a fraction of cells, the hMOR-1AΔ (µ3-like) receptor was excluded from the nucleus and associated with intracellular structures (Figure 4, panels A, B and C). Because the intracellular structures visually resembled endoplasmic reticulum (ER), cells were fixed in paraformaldehyde and stained with an antibody specific for ER. As shown in Figure 5, significant overlap was observed between hMOR-1AΔ (µ3-like) localization and ER-staining, strongly suggesting that this truncated receptor variant may be retained at the ER after synthesis.

### Functional characterization of the receptor variants by measuring cAMP levels

Stably transfected HEK 293 cells expressing GFP-tagged hMOR variants were used in an assay for measurements of intracellular cAMP levels in live cells [23]. Binding of ligand to µ opioid receptors usually causes a reduction in adenylyl cyclase activity via release of the α subunit from the heterotrimeric Gα_i/o_-protein complex, and thereby reduced intracellular levels of cAMP. All 7TM variants (hMOR-1, hMOR-1A, hMOR-1A2 and hMOR-1Y2) were functionally active and lowered forskolin-induced cAMP levels when exposed to DAMGO, morphine, or M6G (Table 2), and these effects were counteracted by concomitant administration of the opioid antagonist naloxone (data not shown). The potency (IC_50_) and efficacy (max inhibition) of the opioids varied between the different receptor variants. For all opioids tested, the highest efficacy was obtained with the classical hMOR-1 receptor. For hMOR-1Y2 the potencies of the opioids were comparable to those obtained with hMOR-1. For hMOR-1A2, all tested opioids had lower potencies, especially M6G, as compared to what was obtained with the other 7TM variants. None of the 6TM variants (hMOR-1AΔ and µ3) had any detectable effect on forskolin-induced cAMP levels. The δ agonist DPDPE had detectable effect on cAMP levels in cells expressing full-length receptors, but significantly less than observed with the µ specific opioids (data not shown).

**Table 2:** Inhibition of forskolin-stimulated cAMP accumulation by opioids in hMOR-1 variants^a^.

	DAMGO		Morphine		M6G	
	IC50 (nM)	Max. inhib. (%)	IC50 (nM)	Max. inhib. (%)	IC50 (nM)	Max. inhib. (%)
hMOR-1	5.8±2.1	70.8±5.3	10.5±2.8	73.5±5.6	27.3±11.2	70.6±5.2
hMOR-1A2	64.5±15.7	63.0±2.7	89.7±25.7	42.9±2.7	239.9±70.1	49.6±3.7
µ3	ND	ND	ND	ND	ND	ND
hMOR-1A	14.5±3.1	43.1±2.3	45.2±15.7	49.3±3.9	16.1±4.7	48.7±3.0
hMOR-1AΔ (µ3-like)	ND	ND	ND	ND	ND	ND
hMOR-1Y2	5.1±2.1	56.1±9.3	17.3±6.8	64.2±1.6	8.1±3.5	50.5±0.8

a The IC_50_ and maximal inhibition were calculated by nonlinear regression analysis using Prism 4.0, GraphPad Software. Results are means ± S.E.M. of 3–7 independent determinations. **Maximal inhibition**: Maximal inhibition was determined after correcting for different receptor expression levels by western blot analyses (Figure S3). The expression levels of receptor variants were calculated relative to that of hMOR-1 (which was set to 1). Significant differences in maximal inhibition were observed for DAMGO (p<0.05), morphine (p<0.01) and M6G (p<0.01). For maximal inhibition by DAMGO, post hoc Tukey analyses showed that hMOR-1 differed from hMOR-1A (p<0.05). For maximal inhibition by morphine, post hoc Tukey analysis showed that hMOR-1 differed from hMOR-1A2 (p<0.01) and hMOR-1A (p<0.05). For maximal inhibition by M6G, post hoc Tukey analysis showed that hMOR-1 differed from hMOR-1A2 (p<0.05) and hMOR-1A (p<0.05). **IC_50_**: Significant differences of IC_50_ were observed for DAMGO (p<0.0001), morphine (p<0.05) and M6G (p<0.05). For DAMGO, post hoc Tukey analyses showed that the IC_50_ value of hMOR-1 differed from hMOR-1A2 (p<0.001), and hMOR-1A2 differed also from hMOR-1A (p<0.001) and hMOR-1Y2 (p<0.001). For morphine, post hoc Tukey analyses showed that the IC_50_ value of hMOR-1 differed from hMOR-1A2 (p<0.05). For M6G, post hoc Tukey analyses showed that the IC_50_ value of hMOR-1 differed from hMOR-1A2 (p<0.05) and hMOR-1A2 differed also from hMOR-1A (p<0.05) and hMOR-1Y2 (p<0.05). ND: not determinable.

## Discussion

The human *OPRM1* gene undergoes extensive alternative splicing at both its 5′ and 3′ end, resulting in a multitude of transcripts which differ in their exon combinations. The majority of these alternatively spliced transcripts encode 7TM hMOR variants which are transcribed from the main promoter associated with exon 1. However, an increasing number of transcripts encoding short peptide fragments or N-terminally truncated 6TM receptor variants have been identified, and these transcripts are produced from alternate promoters located upstream of exons 11 and 13.

Here we present data supporting the existence of yet another promoter in the *OPRM1* gene, which is located upstream of exon 2 and employed in transcription of at least two differentially spliced 6TM variants, hMOR-1AΔ (µ3-like) and µ3. Luciferase reporter gene assays experimentally confirmed the ability of the sequences upstream of exon 2 to promote transcription in human cells, albeit at low levels. The sequence contains numerous putative binding sites for transcription factors, including several elements conforming to the consensus core promoter PyPyANT/APyPy initiator (Inr) sequence, but the significance of these elements for transcription from this promoter is unclear as no detailed functional characterization of the promoter was carried out. The low level of transcription measured in reporter gene experiments is in agreement with the need for nested PCR in order to detect these truncated transcripts in human brain tissue or in neuroblastoma cell lines, suggesting that they are expressed at very low levels.

Interestingly, the µ3 transcript was present in detectable amounts only in BE(2)-C cells whereas hMOR-1AΔ (µ3-like) could be detected in RNA samples from several brain regions. Both transcripts originate from the E2 promoter, and their only structural difference is the retention of exon 3B in hMOR-1AΔ. Exon 3B is also the only structural difference between hMOR-1A and hMOR-1A2. It is therefore tempting to speculate that the ability of the splicing machinery to recognize the splice sites flanking exon 3B may be important in the control of expression of these hMOR variants, and may contribute to a cell- or tissue-specific expression pattern in human brain.

Although an increasing number of 6TM hMOR variants have been reported, their functional significance remains largely unexplored. When overexpressed in HEK 293 cells, hMOR-1AΔ (µ3-like) and µ3 receptors were not expressed at the plasma membrane but instead accumulated in the intracellular compartment. Co-staining of hMOR-1AΔ (µ3-like) transfected cells with an antibody specific for endoplasmic reticulum (ER) indicated that the receptor in a fraction of cells was retained at the ER. However, intracellular retention does not necessarily imply that the 6TMs receptors are biologically inactive. Gris and co-workers [26] showed that the 6TM hMOR-1K variant, despite being retained intracellularly, was biologically active and led to increased Ca^2+^ levels as well as increased nitric oxide (NO) release upon morphine stimulation. Interestingly, unlike hMOR-1, which couples to the inhibitory Gα_i/o_ complex, hMOR-1K couples to the stimulatory Gα_s_ complex and may function to counteract the cellular actions mediated by 7TM hMOR receptors. However, in our study we were unable to demonstrate any effect of the truncated µ3 and hMOR-1AΔ (µ3-like) receptors on cellular cAMP production after exposure to opioid agonists. Similarly, we could not detect any effect of morphine stimulation on intracellular Ca^2+^ mobilization with these truncated variants using a fluorescence-based GFP-certified calcium assay kit (GFP-Certified FluoForte Calcium Assay Kit, Enzo Life Sciences Inc.) and morphine exposure (10µM) for up to 25 min with continuous data recording (data not shown). It should be noted, however, that Gris *et al*. used different cell types, COS1 and BE(2)-C, to demonstrate functionality, and it is possible that the internalized signaling mechanisms could be different or more easily detectable in these cell types than in stably transfected HEK 293 cells. Truncated variants have also been proposed to bind to and modulate the activity of regular 7TM receptors. Indeed, in a recent study truncated variants were shown to be physiologically important through heterodimerization [27]. In that study the 6TM mMOR-1G receptor produced a high-affinity opioid binding target when co-expressed with the ORL_1_ receptor, but was inactive when expressed alone. Thus, important functional roles of truncated MOR-1 variants are beginning to emerge and call for more thorough investigation into their function and endogenous partners in the future.

We found that the full-length receptors, hMOR-1, hMOR-1A, the novel hMOR-1A2, and hMOR-1Y2, accumulated in the plasma membrane, although hMOR-1A2 showed some degree of intracellular retention in a majority of cells. All these variants share identical protein structures across the membrane spanning regions, but differ at the intracellular C-terminus. In accordance with previous observations with traditional full-length hMOR-1 variants [28]–[31], exposure to the opioid peptide DAMGO resulted in agonist-induced endocytosis of receptors, but notably, not with hMOR-1A2. This was surprising since the predicted binding pockets of all four receptors involve identical TM domains. Thus it appears that the unique intracellular C-terminal amino acid sequence of hMOR-1A2, which is identical to that of the reported opioid peptide insensitive µ3 receptor, influence the response to opioid peptides. Importantly, the inability of hMOR-1A2 to internalize did not compromise its ability to mediate opioid-dependent inhibition of adenylyl cyclase, as cAMP assays clearly demonstrated that hMOR-1A2 responds to DAMGO, as well as to morphine and M6G, although the potencies of these opioids were much lower for hMOR-1A2 than for the other full-length receptor variants. Differences in the C-terminus have been shown to influence both the potency and efficacy of opioids [6]. For example, in the present study the IC_50_ values for M6G varied 30-fold between different full-length receptor variants, M6G being most potent with hMOR-1Y2 and least potent with hMOR-1A2, whereas variation in opioid efficacy between variants was less pronounced.

In summary, we have shown that the *OPRM1* gene harbors an alternative promoter upstream of exon 2, which is utilized to generate at least two differentially spliced hMOR transcripts, µ3 and hMOR-1AΔ (µ3-like). When expressed in HEK 293 cells, both 6TM receptors were defective in trafficking to the plasma membrane and did not respond to exogenous opioid stimulation. We have also identified a full-length version of µ3, denoted hMOR-1A2, which contains exon 1 and which exhibits novel functional properties in that it does not internalize in response to DAMGO, but still is able to mediate downstream signaling. The biological relevance of these hMOR-1 splice variants remains unknown, and need to be explored in further studies in order to unravel their potential role in endogenous and exogenous opioid signaling.

## Supporting Information

Figure S1 **Cloning of the E2 promoter.** A 2.1 kb fragment of intron 1, immediately upstream of exon 2, was amplified using the Int-XmaI-4 and Int1-BglII-4 primers indicated, purified and given 3′A ends before subcloning into the PCR 2.1-TOPO vector (Invitrogen). This vector and pGL3 Basic vector were cut with *Kpn*I/*Bgl*II and the purified 2.1 kb DNA fragment was ligated with the linearized pGL3 vector (Promega). The resulting vector was used for measurements of promoter activity in a reporter assay system measuring luciferase activity.
(PDF)

Figure S2 **Amplification and sequencing of transcripts for hMOR-1AΔ (µ3-like) and µ3. A**) hMOR-1AΔ (µ3-like) was amplified by RT-PCR using primers from intron 1 (Int1_F2 and nested Int_a) along with reverse primers (3C_a and nested 3C_b) from the novel exon 3C in µ3, located 336 bp downstream of exon 3A. RNA from different brain regions as indicated was used as template in separate reactions. The amplified product corresponding to mMOR-1AΔ is indicated by an arrow at the left. Sizes (bp) of relevant bands of the size marker (λ cut by *Pst*I) are shown at the right. **B**) Co-amplification of hMOR-1AΔ (µ3-like) and µ3 transcripts from BE(2)-C cells, using primers from intron 1 (Int1_F2 and nested Int_a) along with reverse primers (3C_a and nested 3C_b). The upper band (1378 bp) corresponds to hMOR-1AΔ (µ3-like) whereas the lower band (1042 bp) corresponds to the µ3 transcript. **C**) Separate amplification of transcripts for hMOR-1AΔ (µ3-like, lane 1) and µ3 (lane 2) using DNA picked from the bands in panel B as template. These amplified products were sequenced and verified to represent hMOR-1AΔ (µ3-like) and µ3, respectively. Both transcripts contained the sequence of the Int1_a primer followed by the 42 subsequent 3′-terminal nucleotides of intron 1 directly linked to exon 2. In hMOR-1AΔ (µ3-like) exon 2 was followed by exon 3A, 3B and 3C, whereas in µ3 the 336 bp exon 3B was spliced out (see panels D–F). **D**) Sequencing of the intron1 – exon 2 border of hMOR-1AΔ (µ3-like). The reverse sequence is shown (red line: exon 2, blue line: intron 1). **E**) In hMOR-1AΔ (µ3-like) exon 3A (red) is spliced to the 336 bp exon 3B (blue), followed by exon 3C (yellow). **F**) In µ3 exon 3A (red) is spliced to exon 3C (yellow).
(DOC)

Figure S3 **Expression levels of different splice variants of hMOR-1 in stably transfected HEK293 cells.** GFP-tagged splice variants were detected in total cell extracts using a GFP-specific antibody. The identity of the bands was confirmed by an antibody against human µ opioid receptor (data not shown). Uneven loading was corrected for by relating the signals obtained with the GFP antibody to signals obtained with an antibody against ß-actin. The relative levels of expression were calculated from four separate western blots, loading from 20 to 60 µg of total protein. The values were calculated relative to the hMOR-1 variant and were found to be as follows: hMOR-1: 1, hMOR-1A2: 0.89, hMOR-1A: 1.35 and hMOR-1Y2: 0.69.
(PDF)

Table S1 **PCR primers with integrated restriction sites for cloning of hMOR-1 variants into the pcDNA3-GFP vector.**
(DOCX)

## References

[1] Pasternak GW, Snyder SH (1975) Identification of novel high affinity opiate receptor binding in rat brain. Nature 253: 563–565. 1117990 10.1038/253563a0

[2] Pasternak GW, Childers SR, Snyder SH (1980) Naloxazone, a long-acting opiate antagonist: effects on analgesia in intact animals and on opiate receptor binding in vitro. J Pharmacol Exp Ther 214: 455–462. 6105201

[3] Pasternak GW, Childers SR, Snyder SH (1980) Opiate analgesia: evidence for mediation by a subpopulation of opiate receptors. Science 208: 514–516. 6245448 10.1126/science.6245448

[4] Wolozin BL, Pasternak GW (1981) Classification of multiple morphine and enkephalin binding sites in the central nervous system. Proc Natl Acad Sci U S A 78: 6181–6185. 6273857 10.1073/pnas.78.10.6181 PMC349002

[5] Hahn EF, Carroll-Buatti M, Pasternak GW (1982) Irreversible opiate agonists and antagonists: the 14-hydroxydihydromorphinone azines. J Neurosci 2: 572–576. 6176696 10.1523/JNEUROSCI.02-05-00572.1982 PMC6564265

[6] Pan L, Xu J, Yu R, Xu MM, Pan YX, et al (2005) Identification and characterization of six new alternatively spliced variants of the human mu opioid receptor gene, Oprm. Neuroscience 133: 209–220. 15893644 10.1016/j.neuroscience.2004.12.033

[7] Pasternak DA, Pan L, Xu J, Yu R, Xu MM, et al (2004) Identification of three new alternatively spliced variants of the rat mu opioid receptor gene: dissociation of affinity and efficacy. J Neurochem 91: 881–890. 15525342 10.1111/j.1471-4159.2004.02767.x

[8] Pan YX, Xu J, Mahurter L, Bolan E, Xu M, et al (2001) Generation of the mu opioid receptor (MOR-1) protein by three new splice variants of the Oprm gene. Proc Natl Acad Sci U S A 98: 14084–14089. 11717463 10.1073/pnas.241296098 PMC61171

[9] Pasternak GW (2004) Multiple opiate receptors: deja vu all over again. Neuropharmacology 47 Suppl 1312–323. 15464147 10.1016/j.neuropharm.2004.07.004

[10] Abbadie C, Pan Y, Drake CT, Pasternak GW (2000) Comparative immunohistochemical distributions of carboxy terminus epitopes from the mu-opioid receptor splice variants MOR-1D, MOR-1 and MOR-1C in the mouse and rat CNS. Neuroscience 100: 141–153. 10996465 10.1016/s0306-4522(00)00248-7

[11] Abbadie C, Pan YX, Pasternak GW (2004) Immunohistochemical study of the expression of exon11-containing mu opioid receptor variants in mouse brain. Neuroscience 127: 419–430. 15262332 10.1016/j.neuroscience.2004.03.033

[12] Abbadie C, Pasternak GW (2001) Differential in vivo internalization of MOR-1 and MOR-1C by morphine. Neuroreport 12: 3069–3072. 11568638 10.1097/00001756-200110080-00017

[13] Koch T, Schulz S, Pfeiffer M, Klutzny M, Schroder H, et al (2001) C-terminal splice variants of the mouse mu-opioid receptor differ in morphine-induced internalization and receptor resensitization. J Biol Chem 276: 31408–31414. 11359768 10.1074/jbc.M100305200

[14] Bolan EA, Pan YX, Pasternak GW (2004) Functional analysis of MOR-1 splice variants of the mouse mu opioid receptor gene Oprm. Synapse 51: 11–18. 14579421 10.1002/syn.10277

[15] Wang JB, Johnson PS, Persico AM, Hawkins AL, Griffin CA, et al (1994) Human mu opiate receptor. cDNA and genomic clones, pharmacologic characterization and chromosomal assignment. FEBS Lett 338: 217–222. 7905839 10.1016/0014-5793(94)80368-4

[16] Pan YX, Xu J, Mahurter L, Xu M, Gilbert AK, et al (2003) Identification and characterization of two new human mu opioid receptor splice variants, hMOR-1O and hMOR-1X. Biochem Biophys Res Commun 301: 1057–1061. 12589820 10.1016/s0006-291x(03)00089-5

[17] Bare LA, Mansson E, Yang D (1994) Expression of two variants of the human mu opioid receptor mRNA in SK-N-SH cells and human brain. FEBS Lett 354: 213–216. 7957926 10.1016/0014-5793(94)01129-x

[18] Xu J, Xu M, Hurd YL, Pasternak GW, Pan YX (2009) Isolation and characterization of new exon 11-associated N-terminal splice variants of the human mu opioid receptor gene. J Neurochem 108: 962–972. 19077058 10.1111/j.1471-4159.2008.05833.x PMC2727151

[19] Shabalina SA, Zaykin DV, Gris P, Ogurtsov AY, Gauthier J, et al (2009) Expansion of the human mu-opioid receptor gene architecture: novel functional variants. Hum Mol Genet 18: 1037–1051. 19103668 10.1093/hmg/ddn439 PMC2649019

[20] Cadet P, Mantione KJ, Stefano GB (2003) Molecular identification and functional expression of mu 3, a novel alternatively spliced variant of the human mu opiate receptor gene. J Immunol 170: 5118–5123. 12734358 10.4049/jimmunol.170.10.5118

[21] Cadet P, Mantione KJ, Zhu W, Kream RM, Sheehan M, et al (2007) A functionally coupled mu3-like opiate receptor/nitric oxide regulatory pathway in human multi-lineage progenitor cells. J Immunol 179: 5839–5844. 17947657 10.4049/jimmunol.179.9.5839

[22] Fricchione G, Zhu W, Cadet P, Mantione KJ, Bromfield E, et al (2008) Identification of endogenous morphine and a mu3-like opiate alkaloid receptor in human brain tissue taken from a patient with intractable complex partial epilepsy. Med Sci Monit 14: CS45–49. 18509280

[23] Thakker DR, Ozsoy HZ, Standifer KM (2003) Assessing opioid regulation of adenylyl cyclase activity in intact cells. Methods Mol Med 84: 29–37. 12703314 10.1385/1-59259-379-8:29

[24] Smale ST, Baltimore D (1989) The “initiator” as a transcription control element. Cell 57: 103–113. 2467742 10.1016/0092-8674(89)90176-1

[25] Javahery R, Khachi A, Lo K, Zenzie-Gregory B, Smale ST (1994) DNA sequence requirements for transcriptional initiator activity in mammalian cells. Mol Cell Biol 14: 116–127. 8264580 10.1128/mcb.14.1.116-127.1994 PMC358362

[26] Gris P, Gauthier J, Cheng P, Gibson DG, Gris D, et al (2010) A novel alternatively spliced isoform of the mu-opioid receptor: functional antagonism. Mol Pain 6: 33. 20525224 10.1186/1744-8069-6-33 PMC2894766

[27] Majumdar S, Grinnell S, Le Rouzic V, Burgman M, Polikar L, et al (2011) Truncated G protein-coupled mu opioid receptor MOR-1 splice variants are targets for highly potent opioid analgesics lacking side effects. Proc Natl Acad Sci U S A 108: 19778–19783. 22106286 10.1073/pnas.1115231108 PMC3241767

[28] Arden JR, Segredo V, Wang Z, Lameh J, Sadee W (1995) Phosphorylation and agonist-specific intracellular trafficking of an epitope-tagged mu-opioid receptor expressed in HEK 293 cells. J Neurochem 65: 1636–1645. 7561859 10.1046/j.1471-4159.1995.65041636.x

[29] Keith DE, Murray SR, Zaki PA, Chu PC, Lissin DV, et al (1996) Morphine activates opioid receptors without causing their rapid internalization. J Biol Chem 271: 19021–19024. 8702570 10.1074/jbc.271.32.19021

[30] Burford NT, Tolbert LM, Sadee W (1998) Specific G protein activation and mu-opioid receptor internalization caused by morphine, DAMGO and endomorphin I. Eur J Pharmacol. 342: 123–126. 10.1016/s0014-2999(97)01556-2 9544801

[31] Chen LE, Gao C, Chen J, Xu XJ, Zhou DH, et al (2003) Internalization and recycling of human mu opioid receptors expressed in Sf9 insect cells. Life Sci 73: 115–128. 12726892 10.1016/s0024-3205(03)00250-9

[32] Du YL, Elliot K, Pan YX, Pasternak GW, Inturrisi CE (1997) A splice variant of the mu opioid receptor is present in human SHSY-5Y cells. Soc Neurosci 23: 1206.

[33] Choi HS, Kim CS, Hwang CK, Song KY, Wang W, et al (2006) The opioid ligand binding of human mu-opioid receptor is modulated by novel splice variants of the receptor. Biochem Biophys Res Commun 343: 1132–1140. 16580639 10.1016/j.bbrc.2006.03.084

