# Supplementary figures and images for: The N-Terminally Truncated µ3 and µ3-Like Opioid Receptors Are Transcribed from a Novel Promoter Upstream of Exon 2 in the Human OPRM1 Gene
Source: PLoS One. 2013 Aug 12;8(8):e71024. doi: 10.1371/journal.pone.0071024 (PMC3741380; doi:10.1371/journal.pone.0071024)

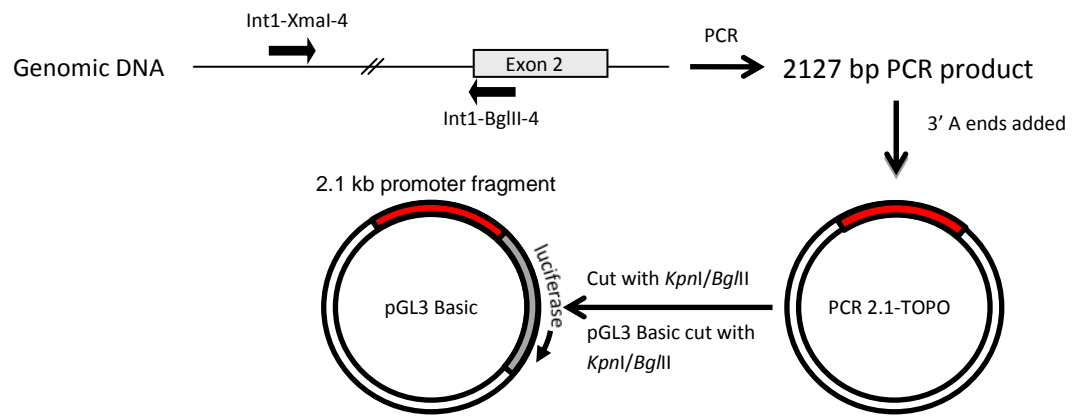

Supplement: Figure S1 — Cloning of the E2 promoter. A 2.1 kb fragment of intron 1, immediately upstream of exon 2, was amplified using the Int-XmaI-4 and Int1-BglII-4 primers indicated, purified and given 3′A ends before subcloning into the PCR 2.1-TOPO vector (Invitrogen). This vector and pGL3 Basic vector were cut with KpnI/BglII and the purified 2.1 kb DNA fragment was ligated with the linearized pGL3 vector (Promega). The resulting vector was used for measurements of promoter activity in a reporter assay system measuring luciferase activity. (PDF) [file pone.0071024.s001.pdf]

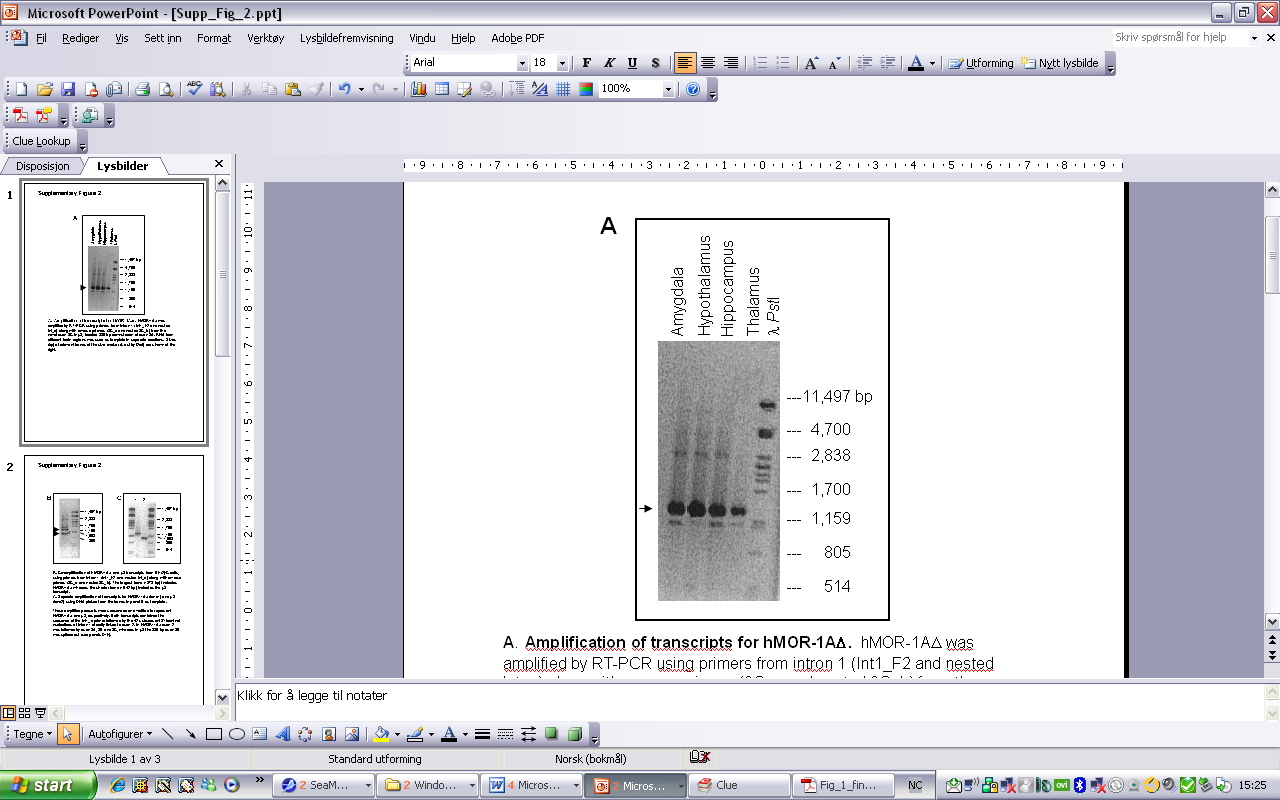


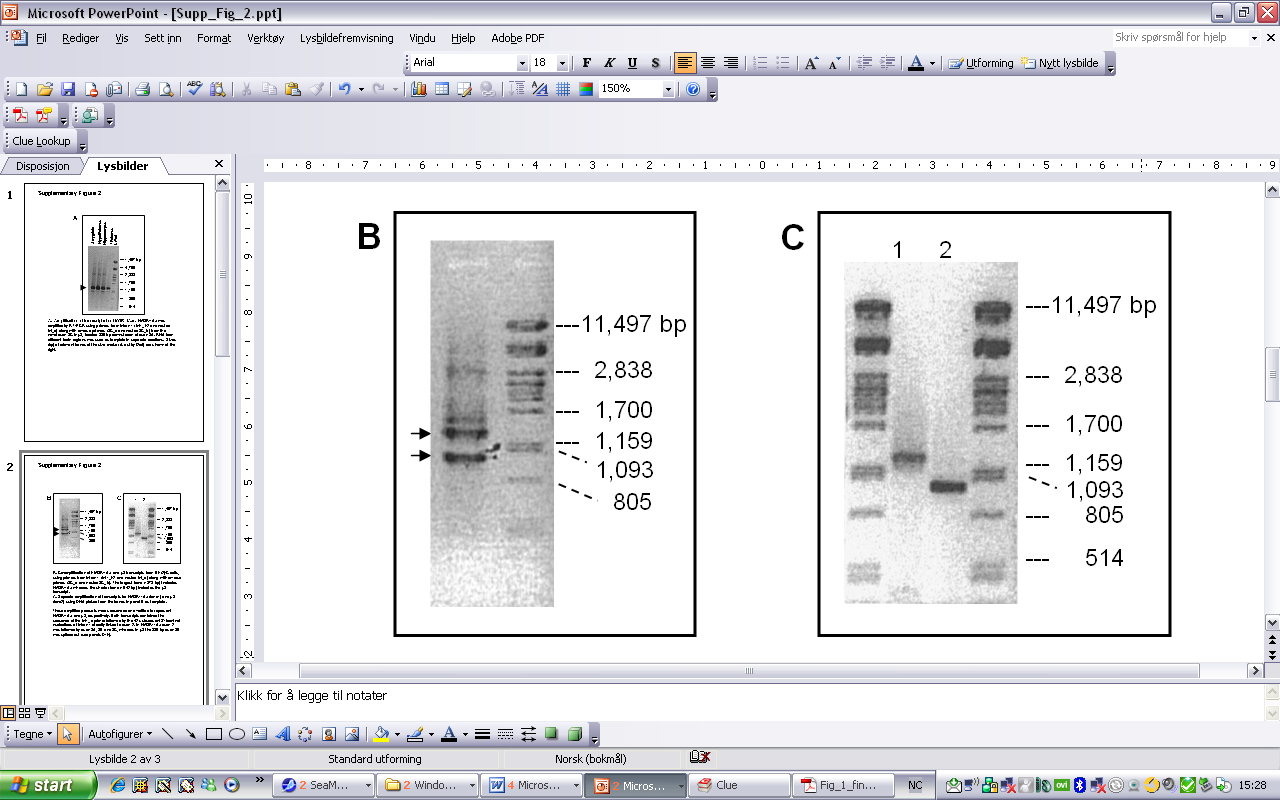


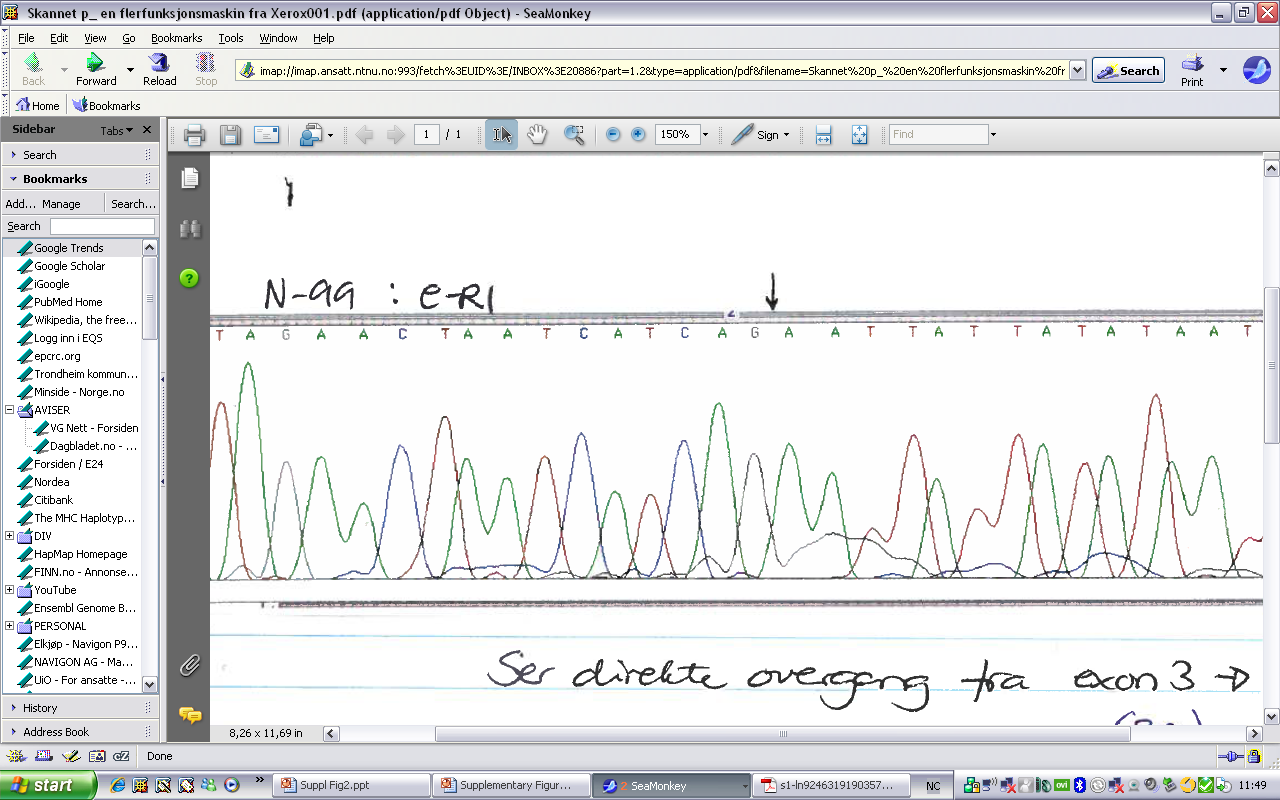


exon 3A – exon 3C

µ3

**F**

**D**


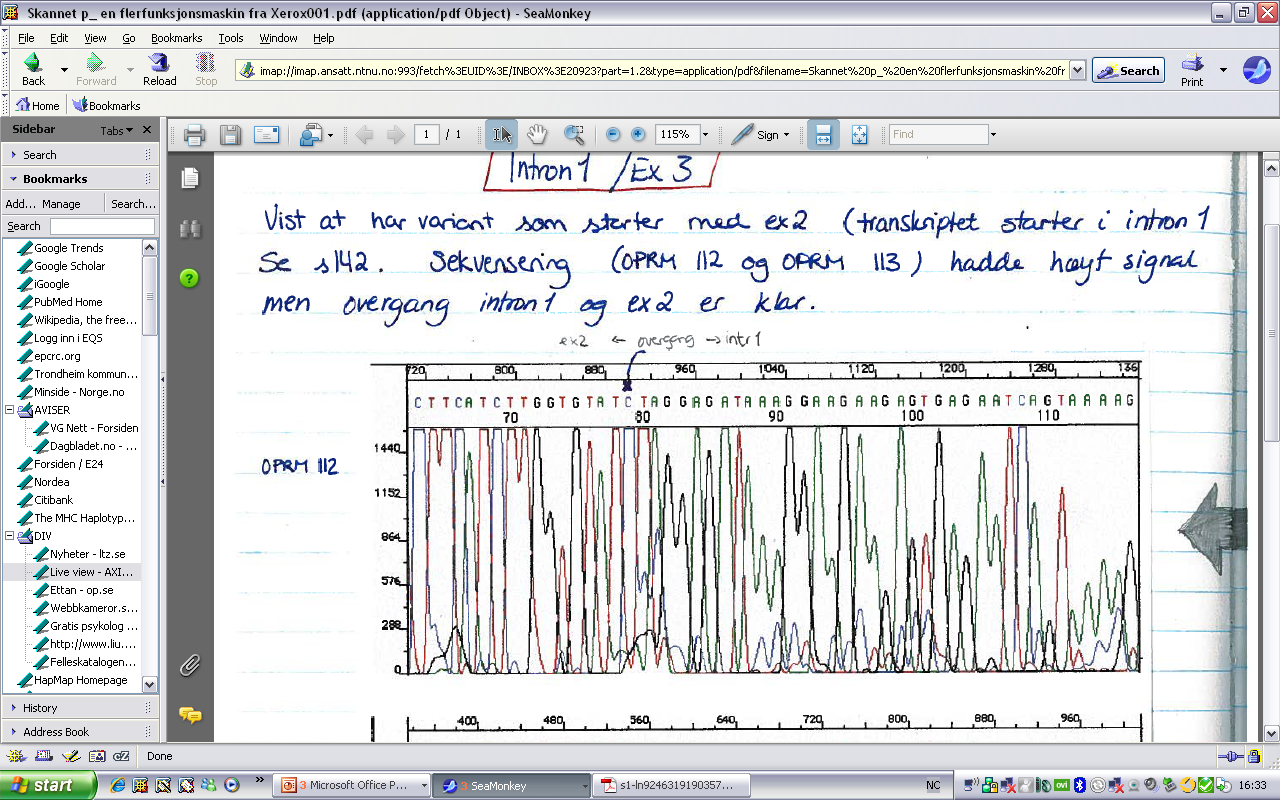


hMOR-1A∆, reverse exon 2 – intron 1 ))))11sequence)

**E**


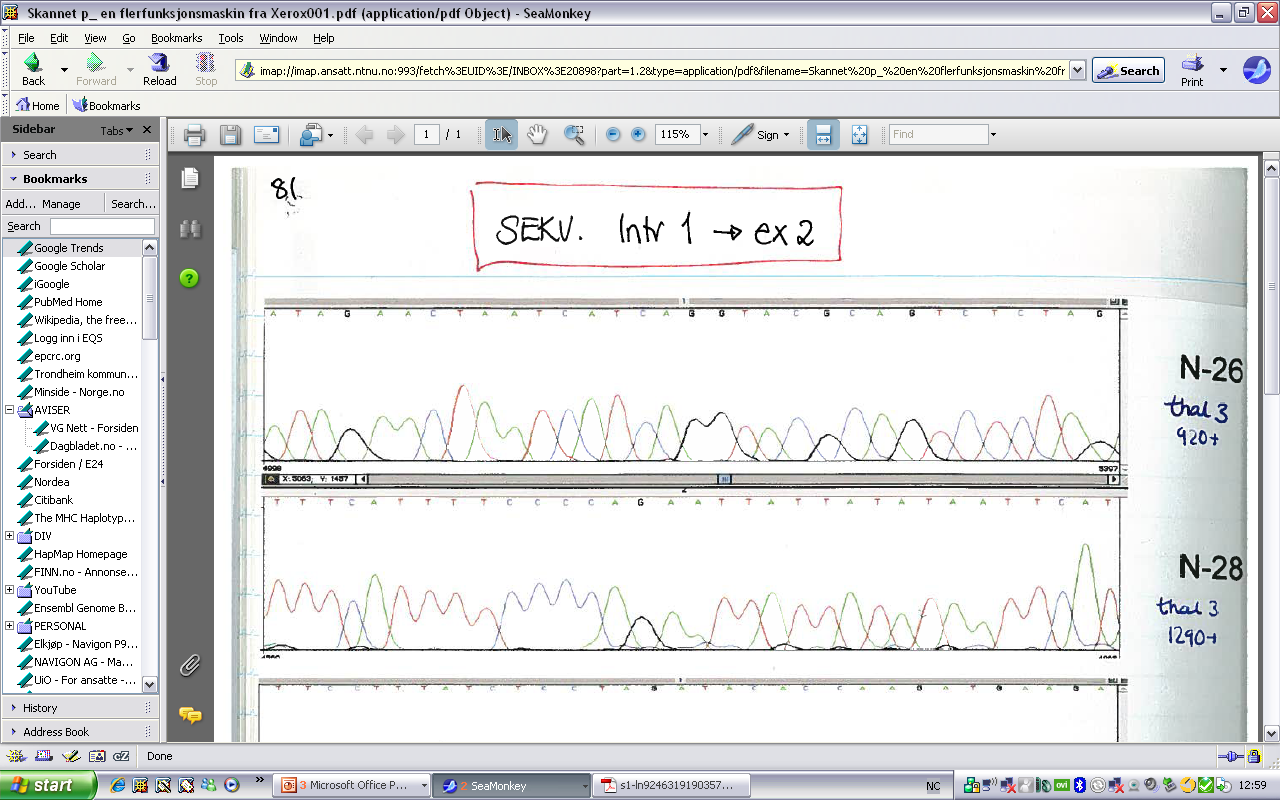


exon 3A – exon 3B

exon 3B – exon 3C

hMOR-1A∆

Supplement: Figure S2 — Amplification and sequencing of transcripts for hMOR-1AΔ (µ3-like) and µ3. A) hMOR-1AΔ (µ3-like) was amplified by RT-PCR using primers from intron 1 (Int1_F2 and nested Int_a) along with reverse primers (3C_a and nested 3C_b) from the novel exon 3C in µ3, located 336 bp downstream of exon 3A. RNA from different brain regions as indicated was used as template in separate reactions. The amplified product corresponding to mMOR-1AΔ is indicated by an arrow at the left. Sizes (bp) of relevant bands of the size marker (λ cut by PstI) are shown at the right. B) Co-amplification of hMOR-1AΔ (µ3-like) and µ3 transcripts from BE(2)-C cells, using primers from intron 1 (Int1_F2 and nested Int_a) along with reverse primers (3C_a and nested 3C_b). The upper band (1378 bp) corresponds to hMOR-1AΔ (µ3-like) whereas the lower band (1042 bp) corresponds to the µ3 transcript. C) Separate amplification of transcripts for hMOR-1AΔ (µ3-like, lane 1) and µ3 (lane 2) using DNA picked from the bands in panel B as template. These amplified products were sequenced and verified to represent hMOR-1AΔ (µ3-like) and µ3, respectively. Both transcripts contained the sequence of the Int1_a primer followed by the 42 subsequent 3′-terminal nucleotides of intron 1 directly linked to exon 2. In hMOR-1AΔ (µ3-like) exon 2 was followed by exon 3A, 3B and 3C, whereas in µ3 the 336 bp exon 3B was spliced out (see panels D–F). D) Sequencing of the intron1 – exon 2 border of hMOR-1AΔ (µ3-like). The reverse sequence is shown (red line: exon 2, blue line: intron 1). E) In hMOR-1AΔ (µ3-like) exon 3A (red) is spliced to the 336 bp exon 3B (blue), followed by exon 3C (yellow). F) In µ3 exon 3A (red) is spliced to exon 3C (yellow). (DOC) [file pone.0071024.s002.doc]

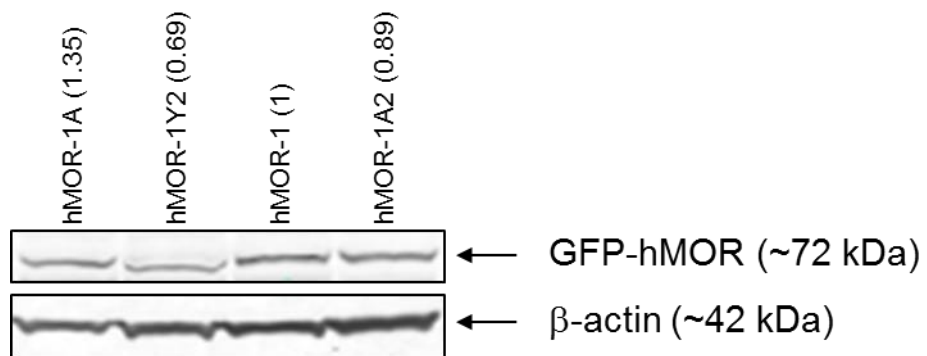

Supplement: Figure S3 — Expression levels of different splice variants of hMOR-1 in stably transfected HEK293 cells. GFP-tagged splice variants were detected in total cell extracts using a GFP-specific antibody. The identity of the bands was confirmed by an antibody against human µ opioid receptor (data not shown). Uneven loading was corrected for by relating the signals obtained with the GFP antibody to signals obtained with an antibody against ß-actin. The relative levels of expression were calculated from four separate western blots, loading from 20 to 60 µg of total protein. The values were calculated relative to the hMOR-1 variant and were found to be as follows: hMOR-1: 1, hMOR-1A2: 0.89, hMOR-1A: 1.35 and hMOR-1Y2: 0.69. (PDF) [file pone.0071024.s003.pdf]
