# Supplementary material for: The N-Terminally Truncated µ3 and µ3-Like Opioid Receptors Are Transcribed from a Novel Promoter Upstream of Exon 2 in the Human OPRM1 Gene
Source: PLoS One. 2013 Aug 12;8(8):e71024. doi: 10.1371/journal.pone.0071024 (PMC3741380; doi:10.1371/journal.pone.0071024)
Supplement: Table S1 — PCR primers with integrated restriction sites for cloning of hMOR-1 variants into the pcDNA3-GFP vector. (DOCX) [file pone.0071024.s004.docx]

**Table S1. PCR primers with integrated restriction sites for cloning of hMOR-1 variants into the pcDNA3-GFP vector**

| **hMOR-1 variant** | **(S/A)** ^††^ | **Sequence** |
| --- | --- | --- |
| hMOR-1 | (S) | 5’-TGCAAGCTTGGCCGTCAGTACCATGGACA-3’ |
|  | (A) | 5’-GACCCTCGAGGGGCAACGGAGCAGTTT-3’ |
| hMOR-1A | (S) | 5’-TGCAAGCTTGGCCGTCAGTACCATGGACA-3’ |
|  | (A) | 5’-AATTCTCGAGACTGCGTACCTGA-3’ |
| hMOR-1A2 | (S) | 5’-TGCAAGCTTGGCCGTCAGTACCATGGACA-3’ |
|  | (A) | 5’- AATTCTCGAGACTGCGTACCTGA-3’ |
| µ3 | (S) | 5’-TTCAAGCTTCTCCTAGATACACCAAGATGAA-3’ |
|  | (A) | 5’-AATTCTCGAGACTGCGTACCTGA-3’ |
| hMOR-1AΔ (µ3-like) | (S) | 5’-TTCAAGCTTCTCCTAGATACACCAAGATGAA-3’ |
|  | (A) | 5’-AATTCTCGAGACTGCGTACCTGA-3’ |
| hMOR-1Y2 | (S) | 5’-TGCAAGCTTGGCCGTCAGTACCATGGACA-3’ |
|  | (A) | 5’-ACAGCTCGAGGAATACAGACACCCTGGGAAG-3’ |

††: S, sense; A, antisense; *Hin*dIII (AAGCTT) and *Xho*I (CTCGAG) sites are underlined
